# Episode of epidemic thunderstorm asthma in Shanxi China September 2025: epidemiological characteristics, mechanisms, and emergency management strategies

**DOI:** 10.3389/fmed.2026.1953484

**Published:** 2026-10-16

**Authors:** Tingting Shen, Guiying Guo, HongMei Li, Junfang Wang, Hongwei Wang, Jianghui Mi

**Affiliations:** 1 Shanxi Provincial Integrated Traditional Chinese Medicine and Western Medicine Hospital, Taiyuan, Shanxi, China; 2 Shanxi University of Chinese Medicine, Jinzhong, Shanxi, China

**Keywords:** allergy, characteristics, emergency department, management, thunderstorm asthma

## Abstract

**Background:**

In September 2025, our emergency department (ED) experienced a sudden influx of 39 asthmatic patients following a thunderstorm. Based on our data, we summarized demographic features of the 39 patients, reviewed literature to describe thunderstorm asthma pathogenesis, and compiled patients’ emergency management processes.

**Method:**

This was a retrospective observational study. Clinical data were collected from electronic medical records and subsequent follow-up. Data were summarized and analyzed using descriptive statistics.

**Results:**

Following standardized management, all patients were successfully managed, ensuring their safety. In this study, male patients accounted for a slightly higher proportion than females. The mean age of the 39 patients was 31.62 ± 12.86 years. The vast majority of patients had a history of allergic rhinitis. Laboratory tests revealed elevated peripheral blood eosinophil counts in some patients. The primary medications administered for thunderstorm asthma included inhaled corticosteroid, short-acting muscarinic antagonists, and short-acting *β*_2_-adrenergic agonist.

**Discussion:**

This study indicates that thunderstorm-related asthma in northern China predominantly occurs in early autumn, with Artemisia pollen as the major allergen. In terms of pathogenesis, thunderstorms induce rupture of Artemisia pollen and release tiny allergenic particles that are inhaled into the airway. Antigens are presented by dendritic cells to activate CD4⁺, Th2 cells, which secrete cytokines such as IL-4 and initiate IgE-mediated allergic inflammation, thereby triggering acute asthma attacks. Meteorological early warning and preventive strategies, as well as hospital-based specialized emergency systems are required.

## 1 Introduction

Thunderstorm asthma lacks a universally standardized definition but is widely recognized as a sudden increase in asthma attacks or bronchospasm symptoms, such as coughing and dyspnea, following a thunderstorm, resulting from the interaction between thunderstorm conditions and aeroallergens (1). The first documented outbreak was reported in Birmingham, UK, in 1983, affecting 106 individuals. Since then, several incidents have been reported across the globe, including in Australia, Europe, the Middle East, and China. The most severe incident occurred in Melbourne, Australia, on November 21, 2016, resulting in over 1,000 people flocking into the emergency room, among them, 500 individuals were hospitalized (2). Although the incidence of thunderstorm asthma is generally low, leading to it being often overlooked, it represents a potential threat to public health and can impose a substantial socioeconomic burden. A large-scale outbreak can overwhelm healthcare systems with unpredictable consequences.

From 20:00 on September 8, 2025, to 03:00 the next day, the ED of Shanxi Provincial Integrated Traditional Chinese Medicine And Western Medicine Hospital treated 39 patients for asthma aggravation. All patients sought medical care within 4 h of a thunderstorm with high winds.

## 2 Method

This was a retrospective observational study. A total of 39 patients with thunderstorm related asthma presenting on the night of the outbreak were enrolled. Clinical data were collected from electronic medical records and subsequent follow-up. Data were summarized and analyzed using descriptive statistics.

## 3 Result

### 3.1 Epidemiological characteristics

The demographic and clinical characteristics of these 39 patients are summarized in Table 1. The present study found that among patients with thunderstorm asthma, males were also slightly more numerous than females. In terms of age composition, we calculated the mean age of these patients to be 31.62 ± 12.86 years. Through a review of patients’ medical histories, we found that 94.87% of the patients had allergic rhinitis. Regrettably, we only performed blood tests on 8 patients with thunderstorm asthma, among these 8 patients, 5(62.5%) exhibited elevated peripheral blood eosinophil counts.

**Table 1:** Patient characteristics.

Characteristic	Categories	Value
Age (*n* = 39, year)		31.62 ± 12.86
Gender (*n* = 39)	Male	20 (51.28)
	Female	19 (48.72)
Allergy history (*n* = 39)	Y	37 (94.87)
peripheral blood eosinophil count (*n* = 8)	>0.52 × 10^9^/L	5 (62.50)

### 3.2 Emergency management strategies

Although rare and seasonal, thunderstorm asthma outbreaks can place immense pressure on healthcare systems, particularly emergency departments. While no universally standardized treatment protocol exists, management primarily involves bronchodilators, glucorticosteroids, public environmental warnings, and health education (3). Our ED faced a rapid surge of patients presenting with consecutive symptoms of chest tightness, cough, wheezing, and dyspnea. To manage the rapidly increasing number of patients effectively while ensuring their safety, we activated our contingency plan, which included recalling off-duty first-line medical staff. All treatments were administered in accordance with established thunderstorm asthma management methods (3, 4). Upon arrival, life-threatening cases would require immediate resuscitation. Fortunately, none of the 39 patients in this series developed life-threatening conditions.

During treatment, the most commonly administered nebulized medications were budesonide inhalation suspension, terbutaline sulfate inhalation solution, and ipratropium bromide inhalation solution. For more severe cases, methylprednisolone sodium succinate for injection or formoterol was administered. Details regarding medication administration are presented in Table 2.

**Table 2:** Medications in thunderstorm asthma patients (*n* = 39).

Medication	Number of users	Percentage (%)
budesonide inhalation suspension	4 (*n* = 39)	10.26
budesonide inhalation suspension and terbutaline sulfate inhalation solution	23 (*n* = 39)	58.97
budesonide inhalation suspension and ipratropium bromide inhalation solution	10 (*n* = 39)	25.64
methylprednisolone sodium succinate for injection	2 (*n* = 39)	5.13
formoterol	4 (*n* = 39)	10.26
Montelukast	32 (*n* = 39)	82.05

One month later, the patients were followed up by telephone, reported no recurrence-related discomfort after discharge, and gained an understanding of the disease and its preventive measures.

## 4 Discussion

The most commonly implicated allergens include grass pollen, olive pollen, and fungal spores (5, 6). During a thunderstorm, strong winds carry allergenic pollen grains into the humid cloud layer, where they can absorb moisture and rupture, releasing allergen-carrying particles ranging from 0.03 to 4 μm in size (7). These micro-particles are then carried to ground level by downdrafts, creating a high concentration of respirable allergens at ground level, which can trigger asthma attacks (8). Furthermore, the high concentration of positive ions in the air during thunderstorms may interact with negatively charged pollen grains, potentially facilitating their rupture and increasing airborne allergen concentrations (9). Studies have confirmed a significant increase in the number of these fragmented pollen particles after thunderstorms (10). In Northern China, the early autumn is the peak season for Artemisia pollen, which can interact with thunderstorm conditions to form the respirable particles that trigger asthma. The precise pathogenesis of thunderstorm asthma is not clearly elucidated. It is hypothesized that allergen-bearing particles smaller than 10 μm can reach the bronchi, triggering an immune response that leads to irritating cough and dyspnea (11). Upon initial exposure to Artemisia pollen particles, the immune system may become sensitized, forming a memory response. Re-exposure leads to the recruitment of mast cells and eosinophils to the lungs, causing an inflammatory reaction and asthma symptoms (12). Dendritic cells, acting as antigen-presenting cells, phagocytose the pollen proteins and process them into fragments recognizable by T cells (13). These antigens are presented to CD4+ T cells via MHC Class II molecules, activating them to differentiate into T-helper 2 (Th2) cells. Activated Th2 cells release cytokines including IL-4, IL-5, and IL-13 (14). IL-4 stimulates goblet cells in the airways to produce excess mucus (15) and promotes B cell differentiation and the production of IgE antibodies. IgE binds to receptors on mast cells, eosinophils, and airway smooth muscle cells. Upon re-exposure, the allergen cross-links the bound IgE, triggering these cells to release various mediators that cause airway smooth muscle contraction and further mucus secretion.

Our study revealed a slightly higher incidence among males. Similar findings have been reported in previous studies, one of which noted that males accounted for up to 58% of cases, exceeding the proportion of females (16). Furthermore, we calculated the mean age of these patients to be 32 years, surprisingly, this value was consistent with the mean age of patients during the 2016 Melbourne thunderstorm asthma event (3). This phenomenon may result from their relatively active immune system. Accordingly, they are more susceptible to thunderstorm asthma symptoms during thunderstorms. Consistently, Nur-Shirin Harun's study also revealed that over 90% of patients with thunderstorm asthma had allergic rhinitis (17). These findings are highly consistent with our results.

For the acute management of these patients, the main nebulized medications included budesonide inhalation suspension, terbutaline sulfate inhalation solution, and ipratropium bromide inhalation solution. Budesonide inhalation suspension is an inhaled corticosteroid(ICS). It exerts anti-inflammatory effects by suppressing the activation of inflammatory cells and the release of allergic mediators, thereby reducing airway hyperresponsiveness and alleviating asthmatic symptoms (18). Terbutaline sulfate is a short-acting *β*_2_-adrenergic agonist (SABA). It exerts a selective agonistic effect on *β*_2_-adrenoceptors expressed on airway smooth muscle, thereby inducing relaxation of bronchial smooth muscle and rapidly relieving acute bronchospasm (19). Ipratropium bromide is classified as a short-acting muscarinic antagonist (SAMA). Via competitive blockade of airway muscarinic receptors, it attenuates vagally mediated bronchoconstriction and reduces hypersecretion of airway mucus (20). Nevertheless, the pharmacological management of thunderstorm asthma still presents certain heterogeneity in clinical practice. Combined application of inhaled medications may help alleviate acute symptoms and reduce the persistence of acute exacerbations. In total, 82.05% of patients received oral montelukast sodium therapy. However, relevant exploration and consensus regarding standardized preventive medication strategies for thunderstorm asthma remain relatively inadequate and require further supplementation.

A review of the literature following this event highlights that after the 2016 Melbourne epidemic, Australia established an environmental monitoring system with a multi-level public alert system disseminated via media. This allows sensitized individuals to take preventive measures, such as staying indoors and using bronchodilators, while alerting healthcare systems to reasonably prepare (3). We recommend that health authorities develop integrated early warning systems combining environmental monitoring and public communication to enhance community preparedness. From another perspective, monitoring instruments targeting allergen concentrations could be engineered to forecast ambient allergen loads and generate early alerts, enabling patients to implement appropriate preventive strategies.

Currently, specific indicators for the diagnosis of thunderstorm asthma remain scarce. Future research is warranted to analyse and summarise laboratory parameters of patients to identify specific diagnostic biomarkers. Exploration of the association between such indicators and prognosis may be carried out when conditions permit, which will assist clinicians in disease diagnosis and prognostic evaluation in clinical work.

According to literature search, thunderstorm asthma outbreaks in northern China are strongly linked to Artemisia species (21). Regrettably, few of our patients underwent allergen detection. A literature search revealed a thunderstorm asthma outbreak reported in Hohhot on September 1st, consistent with the timing in our case (22). These studies indicate that thunderstorm asthma outbreaks in northern China mainly take place in early autumn. Accordingly, we suggest enhancing health education and public science promotion during this season to prevent thunderstorm asthma.

Patients were strongly advised to seek immediate medical help if breathing difficulties persisted. Finally, hospitals must develop specific thunderstorm asthma emergency response plans. Adequate material reserves form the foundation, but these must be supported by robust training on standardized management protocols and workflows.
